# Reducing levels of Violence in the Psychiatric Intensive Care Unit (PICU) - a multidisciplinary quality improvement project

**DOI:** 10.1192/bjo.2021.562

**Published:** 2021-06-18

**Authors:** Zoe Moore, Lynne Pritchard, llana Hamilton

**Affiliations:** Belfast Health and Social CareTrust

## Abstract

**Aims:**

Our aim: To reduce the number of Level 1* violent incidents in Ward 4 by 30% by April 2020

*Level 1 is defined as “Behaviour involving force, which causes or is intended to cause physical harm to others; but excludes assault on objects, threats or verbal abuse”

Ward 4 is Belfast Health and Social Care Trust's only PICU, with a total of 6 beds. Our project took place on the background of a recent move to a new purpose-built inpatient unit, as well as a trust-wide initiative to address levels of violence across inpatient psychiatry services.

**Method:**

We divided our project into 3 main areas:

Patient factors

Staff factors

Environmental factors

We identified and implemented a number of change ideas, using Plan-Do-Study- Act methodology, regularly meeting to review progress and plotting our data on a run chart.

Key patient interventions included a “Mutual Respect” exercise and regular “Community Meetings”.

Staff interventions included use of Safety Crosses, Daily Safety Briefings and the Broset Violence Checklist (BVC).

Environmental factors were continually assessed and escalated as appropriate.

We raised awareness of our project and gained feedback by creating a dedicated notice board, providing a staff information session and including it as an agenda item at ward meetings.

Our project measures were identified as:

Outcome: Number of level 1 violent incidents occurring per week

Balancing: Number of incidents in other categories; Patient satisfaction

Process: Staff safety rating; Engagement with interventions

**Result:**

Unfortunately, we were unable to meet our initial goal and there continued to be considerable variation in the number of weekly incidents.

We believe this was attributable to several factors, including the level of acuity within the ward during the project timeframe. It was noted that a relatively small number of patients contributed to a large proportion of the total incidents. Our results, therefore, did not reflect the success of interventions with other patients on the ward.

Despite this, we noted improvements in terms of patient and staff engagement with the project, including subjective reports of staff safety during shifts.

**Conclusion:**

The unpredictable and complex nature of the PICU setting cannot be under-estimated and this ultimately impacted on achieving our intended outcome.

We do feel, however, that the project has had a positive impact and we hope we can build on this progress over the coming months.

Further interventions are being explored, including personalised daily activity schedules and attempts to reduce levels of continuous observations.

